# A Microwave-Assisted Synthesis of Zinc Oxide Nanocrystals Finely Tuned for Biological Applications

**DOI:** 10.3390/nano9020212

**Published:** 2019-02-06

**Authors:** Nadia Garino, Tania Limongi, Bianca Dumontel, Marta Canta, Luisa Racca, Marco Laurenti, Micaela Castellino, Alberto Casu, Andrea Falqui, Valentina Cauda

**Affiliations:** 1 Department of Applied Science and Technology, Politecnico di Torino, Corso Duca degli Abruzzi 24, 10129 Turin, Italy; nadia.garino@polito.it (N.G.); tania.limongi@polito.it (T.L.); bianca.dumontel@polito.it (B.D.); marta.canta@polito.it (M.C.); luisa.racca@polito.it (L.R.); marco.laurenti@polito.it (M.L.); micaela.castellino@polito.it (M.C.); 2 Istituto Italiano di Tecnologia, Center for Sustainable Future Technologies, Via Livorno 60, 10144 Torino, Italy; 3 King Abdullah University of Science and Technology (KAUST), Biological and Engineering (BESE) Division, NABLA Lab, Thuwal 23955, Saudi Arabia; alberto.casu@kaust.edu.sa (A.C.); andrea.falqui@kaust.edu.sa (A.F.)

**Keywords:** zinc oxide, microwave solvothermal synthesis, hydrodynamic size, surface chemistry, nanocrystals, cell cytotoxicity

## Abstract

Herein we report a novel, easy, fast and reliable microwave-assisted synthesis procedure for the preparation of colloidal zinc oxide nanocrystals (ZnO NCs) optimized for biological applications. ZnO NCs are also prepared by a conventional solvo-thermal approach and the properties of the two families of NCs are compared and discussed. All of the NCs are fully characterized in terms of morphological analysis, crystalline structure, chemical composition and optical properties, both as pristine nanomaterials or after amino-propyl group functionalization. Compared to the conventional approach, the novel microwave-derived ZnO NCs demonstrate outstanding colloidal stability in ethanol and water with long shelf-life. Furthermore, together with their more uniform size, shape and chemical surface properties, this long-term colloidal stability also contributes to the highly reproducible data in terms of biocompatibility. Actually, a significantly different biological behavior of the microwave-synthesized ZnO NCs is reported with respect to NCs prepared by the conventional synthesis procedure. In particular, consistent cytotoxicity and highly reproducible cell uptake toward KB cancer cells are measured with the use of microwave-synthesized ZnO NCs, in contrast to the non-reproducible and scattered data obtained with the conventionally-synthesized ones. Thus, we demonstrate how the synthetic route and, as a consequence, the control over all the nanomaterial properties are prominent points to be considered when dealing with the biological world for the achievement of reproducible and reliable results, and how the use of commercially-available and under-characterized nanomaterials should be discouraged in this view.

## 1. Introduction

In the last decade metal oxide semiconducting nanoparticles (NPs) have been receiving great interest in the field of biological applications due to their intriguing optical properties, low toxicity, good biocompatibility and their low cost [1]. Among them, zinc oxide (ZnO) nanoparticles have shown to be particularly promising due to their peculiar chemical and physical properties, which can be specifically tailored on the basis of the particles’ size and shape [2,3]. As an example, the wide bandgap typical of ZnO (about 3.37 eV at room temperature, RT) entails a fluorescence excitation situated in the ultraviolet (UV) region [4], which allows ZnO to be successfully employed for optical cell imaging. More in general, it has been demonstrated how ZnO could be a promising material for therapeutic and diagnostic applications [2], showing high levels of drugs loading and a quite easy control over the following release [5,6]. In this context, the optical, targeting and drug delivery properties of ZnO can be more specifically addressed by the combination of various synthetic procedures (sol-gel, sputtering, hydro-solvothermal, etc.) [7] and ZnO morphologies (nanowires, nanorods, nanobelts, desert roses and spherical nanoparticles) [8,9], together with surface functionalization approaches [2,10]. To this purpose, it is important to observe that crystal density, morphology and defects could be critical factors in determining the material properties, and as a consequence, the final applications. Although ZnO nanostructures featuring dimensions lower than 100 nm are nowadays considered the most promising for biomedicine [11,12,13], the most part of the literature generally neglects the influence of the synthesis processes and parameters on the characteristics of the resulting NPs and, finally, on the reproducibility of their biological response. Actually, this is an essential point for further strengthening the biological application of crystalline nanoparticles. In this regard, it is worth mentioning that many articles deal with the use of commercial particles (with the limit about their morphologies and size distribution) or did not report any specific detail about important synthesis aspects like the synthesis precursors, the surface chemistry, and sometimes the hydrodynamic size and z-potential [14,15].

In this work, we report a novel synthetic approach of ZnO nanocrystals (NCs) based on a microwave-assisted solvothermal process that allows us to reach a greater control over the morphology and dimensional dispersion of the NPs. At the same time this new method guarantees a higher reproducibility level of experimental data with respect to those obtained by using ZnO NCs synthesized with a more conventional wet approach. The microwave-assisted synthesis also presents additional advantages, like shorter reaction time and lower energy consumption. Actually, one of the main characteristics of this technique is to guarantee a uniform heating of the precursors and to present outstanding reaction rates, moving from several hours to a few minutes [16,17,18]. Furthermore, this synthetic method assures high reliability and high reaction yields. This explains the growing popularity and diffusion of microwave-assisted synthetic approaches over a wide range of different nanomaterials synthesis [19,20], but to our knowledge very few reports are dedicated to the preparation of ZnO-based nanomaterials [21,22,23,24]. Indeed, thanks to this synthetic route we obtain a simultaneous nucleation of nanocrystals that leads to a uniform and reproducible dimensional dispersion of ZnO (about 20 nm in diameter), with outstanding colloidal stability both in ethanol and in water. In this way, we demonstrate how the synthetic route should be an important factor to be considered for the achievement of reproducible and reliable results, also when evaluating the cell viability when in contact with these nanocrystals. 

## 2. Materials and Methods 

### 2.1. Synthesis, Functionalization and Labelling of ZnO Nanocrystals.

All the chemicals were used as purchased without further purification. ZnO nanocrystals were synthesized through two different synthetic routes: a traditional solvothermal way (sample named ZnO-st) and a microwave-assisted synthesis (sample named ZnO-mw). As zinc precursors we chose zinc acetate di-hydrate (Zn(CH_3_COO)_2_·2H_2_O Puriss. p.a., ACS Reagent, ≥99.0% Fluka) and a hydroxide as mineralizing agent (Sigma-Aldrich) both dissolved in methanol (Reag. Ph Eur Grade VWR Chemicals). The reaction path, in both cases, is based on the hydrolysis of the zinc precursor due to the presence of the hydroxide, as shown in the following reactions scheme:

KOH → K+ (aq) + OH− (aq)(1)

Zn(Ac)_2_ → Zn^2+^ (aq) + 2Ac^−^ (aq) (2)

Zn^2+^ (aq) + 2OH^−^ (aq) → Zn(OH)_2_ (aq) (3)

Zn(OH)_2_ (aq) + 2H_2_O → Zn(OH)_4_^2−^ + 2H^+^ (aq) (4)

Zn(OH)_4_^2−^ + 2H^+^ (aq) → ZnO (s) + 3H_2_O (5)

The microwave-assisted synthesis was carried out as follows. A solution containing the zinc precursor in methanol (0.09 M, 60 mL) was prepared and stirred directly in the Teflon reactor vessel. In order to better initiate the zinc oxide nucleation, 480 µL of double-distilled water was added and then the potassium hydroxide solution (0.2 M, 35 mL) (KOH ≥ 85% pellets, Sigma-Aldrich) was mixed together in a 270 mL Teflon reactor vessel, equipped with pressure and temperature probes, connected with the microwave furnace (Milestone START-Synth, Milestone Inc, Shelton, Connecticut). The resulting solution was put into a microwave oven for 30 min at 60 °C. After the completion of the reaction, the solution was cooled down to room temperature and followed by two washing steps to change the reaction solvent and to remove any unreacted compound. To do that, the colloidal solution was collected and centrifuged for 10 min at 3500 g (Mega Star 600R, VWR), the supernatant was then removed, and the precipitate was dispersed and washed twice in 15 mL of ethanol (Sigma-Aldrich, 99%). The as-obtained ZnO NCs pellet was suspended through sonication (LABSONIC LBS2, FALC Instruments SRL) in fresh ethanol to give the final colloidal suspension. 

The traditional solvothermal synthesis process was carried out as already reported [25] with the same zinc precursor at the same concentrations in a round-bottom glass flask (100 mL). In detail, zinc acetate di-hydrate (0.09 M) was directly dissolved in the reaction flask with methanol (42 mL) and heated under continuous stirring in reflux conditions since the temperature of 60 °C was reached and the double-distilled water was added (318 µL). The methanol solution of sodium hydroxide (0.31 M, 23 mL) (NaOH BioXtra, ≥98% acidimetric, pellets anhydrous, Sigma-Aldrich) was then added dropwise to the zinc acetate solution (in about 20 min). The reaction conditions were maintained for 2.5 h and after this time the as obtained suspension was cooled to RT. ZnO NCs (named ZnO-st) were collected and washed with fresh ethanol (Sigma-Aldrich, 99%) as previously reported for the microwave-assisted synthesis. Reaction yields were evaluated for both the synthetic procedures by weighing the dried NCs from a known volume of the obtained colloidal solutions. 

Both the typologies of as synthesized ZnO NCs were functionalized in order to proceed with the in vitro cell culture studies, according to a previously reported method [25,26]. In particular, the ZnO NCs surface was decorated with the amino-propyl group (ZnO-NH_2_ NCs). Approximately 50 mg of ZnO NCs, dispersed in 20 mL of ethanol (Sigma-Aldrich 99%), were heated to 80 °C in a 25 mL round glass flask under continuous stirring and nitrogen gas flow. A 10 mol% ratio of 3-aminopropyltrimethoxysilane (H_2_N(CH_2_)_3_Si(OCH_3_)_3_ APTMS 97%, Sigma Aldrich, 10 μL), with respect to total ZnO amount, was added to the NCs suspension and the reaction was carried out for 6 h. The excess of unreacted APTMS was then removed by washing twice the ZnO NCs with fresh ethanol, separating them from the reaction medium by centrifugation (10 min, 10,000 g).

Only for the internalization of nanocrystals into cancer cells, the ZnO-NH_2_ NCs were coupled with ATTO633-NHS ester dyes (Thermofischer), by adding 2 μg of dye each mg of NCs in ethanol suspension. The as obtained solution was dark-stirred overnight and then washed twice by centrifuging (10 min, 10,000 g) and resuspending the pellet in fresh ethanol to remove unbounded dye molecules [25]. To minimize the effects of particles aggregation and sedimentation under biological tests, the suspension of dye-labelled nanocrystals was always freshly prepared and shortly sonicated (10 min) before each experiment.

### 2.2. Characterization

The crystalline structure of the prepared materials was investigated by XRD (X-ray diffraction). A Panalytical X’Pert diffractometer in θ–2θ Bragg-Brentano configuration equipped with a source of radiation Cu-Kα (λ = 1.54 Å, 40 kV and 30 mA) was employed. The samples were prepared depositing the colloidal solution drop by drop on a silicon wafer. The XRD analysis was carried out at room temperature in the 2θ range 20°–65° with a step size of 0.02° (2θ), and an acquisition time of 100 s per step.

Further information on the chemical composition and surface of the produced materials was provided by X-ray photoelectron spectroscopy (XPS) analysis. A PHI 5000 Versaprobe Scanning X-ray photoelectron spectrometer (monochromatic Al K-alpha X-ray source with 1486.6 eV energy) was used to investigate the material chemical composition. A spot size of 100 μm was used in order to collect the photoelectron signal for both the high resolution (HR) and the survey spectra.

Field emission scanning electron microscopy (FESEM, Merlin, ZEISS, Jena, Germany) was used to evaluate the overall quality and the morphology of the different materials. The samples were prepared by depositing a drop of a properly diluted NCs solution on top of silicon wafer.

Both conventional and high-resolution transmission electron microscopy (CTEM and HRTEM) were used to characterize the morphological and structural features of the different materials. CTEM was performed by using a FEI Tecnai Spirit microscope working at an acceleration voltage of 120 kV, equipped with a Twin objective lens, a LaB_6_ thermionic electron source and a Gatan Orius CCD camera. HRTEM was performed by using a FEI Titan ST microscope working at an acceleration voltage of 300 kV, equipped with a S-Twin objective lens, an ultra-bright field emission electron source (X-FEG) and a Gatan 2 k × 2 k CCD camera.

The hydrodynamic size of the nanocrystals in ethanol and water was determined using the dynamic light scattering (DLS) technique with a Zetasizer Nano ZS90 (Malvern Instruments, Worcestershire, UK). All the measurements were performed at room temperature, at a concentration of 100 µg/mL sonicating each sample for 10 min before the acquisition.

UV−visible spectra were recorded in absorbance with a Multiskan GO microplate UV−Vis spectrophotometer (Thermofisher Scientific) using the ZnO NCs in ethanolic suspension at a concentration of 0.5 mg/mL. All the spectra were background subtracted.

Fluorescence excitation and emission spectra were recorded by a Perkin Elmer LS55 fluorescence spectrometer (PerkinElmer Inc. Waltham, Massachusetts, MA, USA) using quartz cuvettes of 1 cm optical path and containing the ZnO NCs in ethanolic suspension at a concentration of 0.5 mg/mL. For the emission spectra, the fluorescence excitation wavelength used was 380 nm and, for the excitation spectra, the fluorescence emission wavelength was 500 nm. Scans were acquired with 2.5 slits opening and at a scan rate of 300 nm/min. 

### 2.3. In Vitro Mammalian Cell Culture Biological Test

Cell culture and reagents. KB cell line (ATCC^®^ CCL17TM) was purchased by the American Type Culture Collection. Cells were grown in minimal essential Eagle’s medium (EMEM, Sigma) supplemented with 10% heath inactivated fetal bovine serum (FBS, Sigma), 100 units/mL penicillin and 100 µg/mL streptomycin (Sigma) and maintained at 37 °C under a 5% CO_2_ atmosphere. Cells were periodically tested for mycoplasma infection.

All the NCs solutions were freshly prepared from 1 mg/mL ethanol stock solutions. The ZnO NCs were bath sonicated at 40 kHz, 100% power for 10 min before being added to the culture medium and then immediately used for treating cells.

Cytotoxicity tests. 1.5 × 10^3^ cells/well were seeded onto 96-well plastic culture plates (Corning^®^ 96 Well TC-Treated Microplates) and incubated at 37 °C, 5% CO_2_. After 24 h the cell medium was replaced with fresh medium containing ZnO-st-NH_2_ or ZnO-mw-NH_2_ at different concentrations (10, 15, 20, 25 µg/mL). After 24 h of incubation, cell proliferation was assessed by measuring cell metabolic activity through the WST-1 cell proliferation assay. 10 µL of the WST-1 reagent (Roche) were added to each well and after 2 h incubation in the dark at 37 °C, 5% CO_2_, the formazan absorbance was measured at 490 nm by the Multiskan GO microplate spectrophotometer (Thermofisher Scientific) using a 620 nm reference. Control values (without treatments) were set at 100% viable and all values were expressed as a percentage of the control. The inhibitory concentration 50% (IC50) of the ZnO-mw-NH_2_ was determined online using the IC50 calculator tool of the AAT Bioquest webpage [27].

Cell internalization. NCs internalization in KB cells was measured using a Guava Easycyte 6-2L flow cytometer (Merck Millipore). 1 × 10^5^ cells/well were seeded onto a 6-well plate (Corning^®^ Costar^®^ TC-Treated) with complete cell culture medium 24 h prior the assay. Then, cells were treated with 10 µg/mL of ZnO-st-NH_2_ or ZnO-mw-NH_2_ labeled with ATTO633-NHS ester dye (Thermofisher). A control well, containing the untreated cells, was instead filled with fresh medium without NCs. After 24 h incubation, cells were rinsed twice with phosphate buffered saline (PBS), trypsinized and centrifuged at 130 g for 5 min. Cell pellets were re-suspended in 1 mL PBS and immediately analysed with the flow cytometer; 10,000 gated events were considered for the analysis excluding cellular debris, characterized by low FSC (forward scatter) and SSC (Side scatter). Results were reported as the percentage of fluorescence positive events, characterized by a shift in fluorescence intensity compared to untreated cells. This evaluation was performed by Guava InCyte Software (Merck Millipore, Darmstadt, Germany). Independent experiments were performed 3 times. The different NCs internalization was compared using a t test and when equal variance test failed, Welch’s test was assumed instead of Student’s t test.

Statistical analysis. All experiments were done at least in triplicate and the results were presented as mean ± SEM (standard error of mean). The experimental data were analysed using Sigmaplot software version 14, demo version (Systat Software Inc., San Jose, California, CA, USA). t test and One Way Analysis of Variance were executed, where equal variances were not assumed, the results of Welch’s test were presented. When the normality test failed, Mann–Whitney rank sum test was run. Differences were considered significant at *p* value < 0.05. 

## 3. Results and Discussion

Both synthetic approaches allowed us to obtain milky-coloured colloidal suspensions of ZnO NCs in ethanol. The yields comparison shows that larger yields (i.e., five times higher) are obtained for the microwave assisted procedure with respect to the traditional wet route. The functionalization with amino-propyl groups provides on the one hand a positively charged surface, useful to improve the colloidal stability of the NCs in solution. On the other hand, the amine groups are an ideal anchoring site for dye labelling, commonly used for fluorescence microscopy experiments, as well as for the further functionalization with polymers, lipids or biomolecules useful to enhance the interaction with cells and biological fluids [25,26].

### 3.1. Morphological and Structural Characterization of ZnO Nanocrystals (NCs)

**Figure 1:**
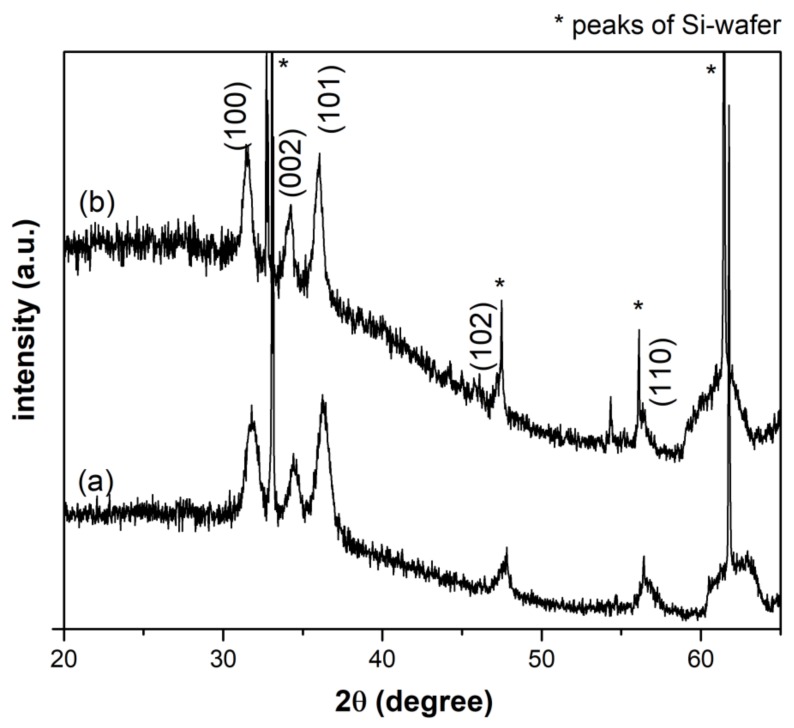
X-ray diffractogram of the pristine ZnO nanocrystals (NCs) obtained via (**a**) conventional (ZnO-st NCs) and (**b**) microwave-assisted synthesis (ZnO-mw NCs).

XRD is performed in order to obtain information about phase identification and quantification, percentage of crystallinity, crystallite size and unit cell size. The XRD patterns of pristine ZnO NCs from both synthetic routes are reported in Figure 1. The comparison of the diffractograms with the standard XRD pattern of ZnO (JCPDS card n. 36-1451) confirms the crystalline structure of the particles. In particular, the peaks at 31.9°, 34.4°, 36.4°, 47.6°, 56.7°, 62.9° are indexed to (100), (002), (101), (102), (110) and (103) planes, respectively, which corresponds to the Miller index of typical hexagonal wurtzite structure. The strongest reflection (101) of each XRD pattern was considered to estimate the average crystallites size with the Debye–Scherrer equation, obtaining a mean diameter of 10.5 nm for ZnO-st NCs and 15.5 nm for ZnO-mw NCs, respectively.

**Figure 2:**
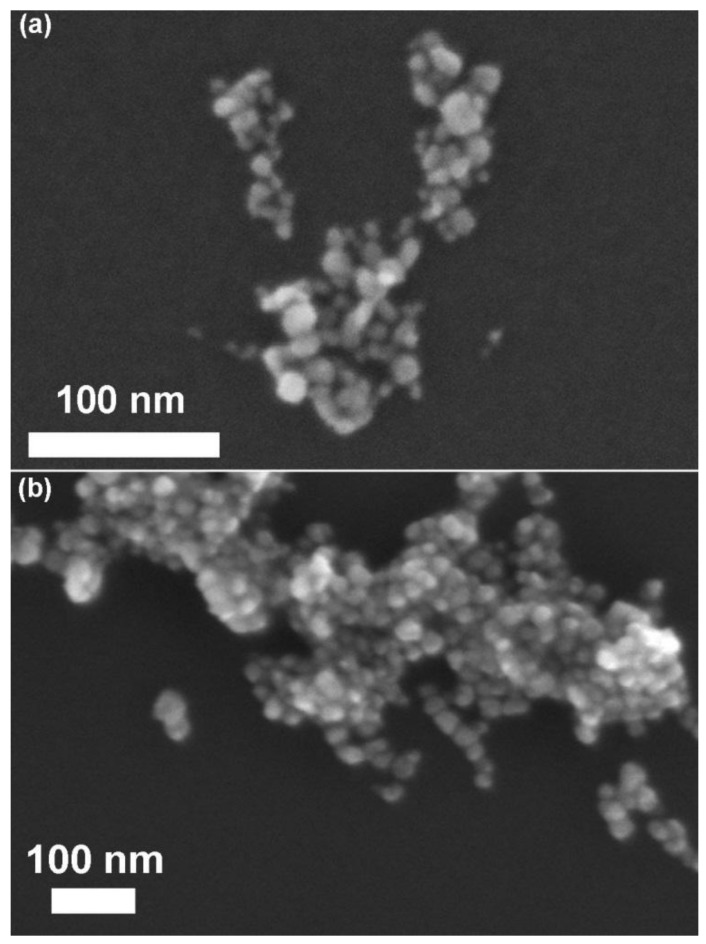
Field emission scanning electron microscopy (FESEM) images of pristine ZnO NCs obtained via (**a**) conventional synthesis and (**b**) microwave-assisted route.

In order to evaluate the synthesis’ repeatability, all the samples are actually characterized by comparing different batches obtained by the two synthetic procedures. FESEM images (here reported in Figure 2) show ZnO spherical morphology for the microwave synthetic route, whereas different shapes, even some elongated ones, are observed for the conventionally-synthesized NCs. By comparing dimensional measurements, we note that the NPs obtained via microwave-assisted synthesis highlight a narrower and reproducible range of particle size distribution, with respect to the traditional one. Indeed, the size distribution of ZnO-mw NCs presents an average size of 20 nm (±5 nm), while for ZnO-st NCs the dimensional range varies between 6 and 20 nm.

**Figure 3:**
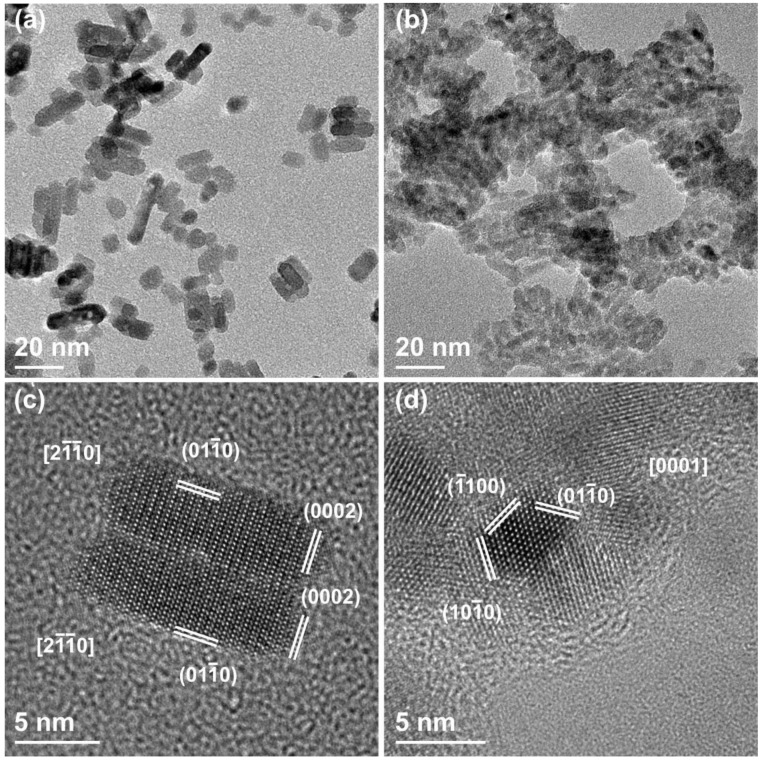
(**a**,**b**) Conventional transmission electron microscopy (CTEM) and (**c**,**d**) high-resolution transmission electron microscopy (HRTEM) images of (**a**,**c**) pristine ZnO-st NCs and (**b**,**d**) functionalized ZnO-st-NH_2_, both obtained via solvothermal synthesis.

Transmission electron microscopy is also carried out to investigate the structure and crystallinity of ZnO and ZnO-NH_2_ nanocrystals synthesized with both methods. Thanks to the higher magnification and resolution, it is possible to better highlight the differences between the two as-obtained NC populations. The ZnO-st NCs are reported in Figure 3, where panels (a) and (c) show the CTEM and HRTEM images of the pristine (not functionalized) ZnO-st sample. These particles have a short rod-like shape, whose length goes from 7 to 40 nm and an almost constant width, around 7–8 nm. Besides, HRTEM clearly indicates that these rod-like structures have monocrystalline nature, with no evidence of defects, and with lattice sets’ d-spacing and corresponding angular distances expected for the wurtzite structure of ZnO. Panel (b) and (d) of the Figure 3 show what happens to the same ZnO-st particles after functionalization with the amino-propyl groups. Two apparent differences are observed with respect to the non-functionalized crystals: (i) the nanoparticles tend to aggregate, and (ii) the clear presence of an amorphous shell is noticed, surrounding the NCs and clustering them. Again, the HRTEM analysis shows the wurtzite hexagonal structure expected for the ZnO.

**Figure 4:**
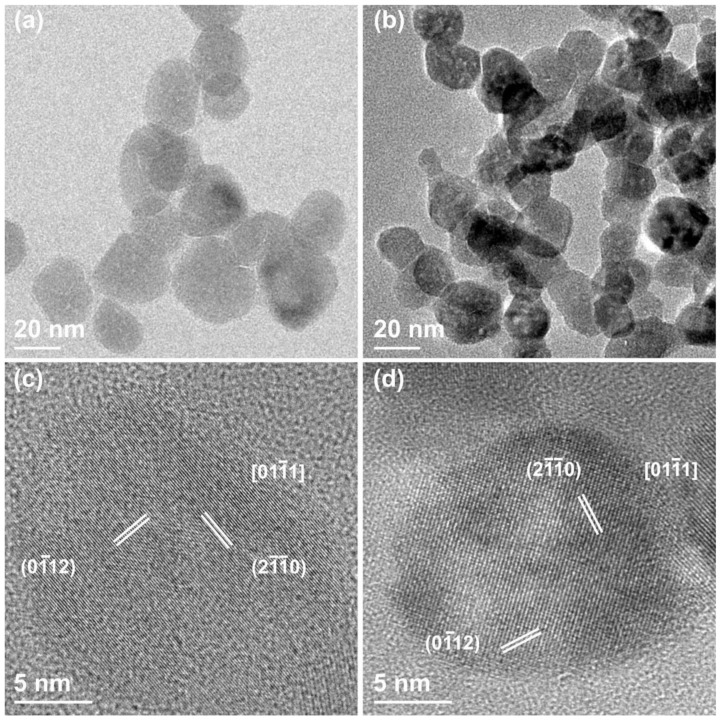
(**a**,**b**) CTEM and (**c**,**d**) HRTEM images of (**a**,**c**) pristine ZnO-mw NCs and (**b**,**d**) functionalized ZnO-mw-NH_2_, both obtained via microwave-assisted synthesis.

Figure 4 displays the ZnO-mw nanocrystals where panels (a) and (c) show the pristine NCs, and panels (b) and (d) those functionalized with the amino-propyl groups. When comparing these images to the two groups prepared by the traditional solvothermal synthesis and displayed in Figure 3, some important differences and analogies have to be highlighted for both. First, the ZnO-mw NCs show a spherical, often faceted, morphology. Their size ranges from 15 to 25 nm, confirming the results obtained by FESEM characterization. HRTEM indicates that both the pristine and the functionalized nanocrystals have the wurtzite hexagonal monocrystalline structure with lattice sets d-spacing and corresponding angular distances expected for the zinc oxide material. Finally, as in the case of the functionalized ZnO-st NCs, no difference in terms of size can be observed between the pristine and amine-functionalized ZnO-mw ones, with the latter displaying an evident propensity to clustering as well as the presence of an amorphous external shell surrounding them.

**Figure 5:**
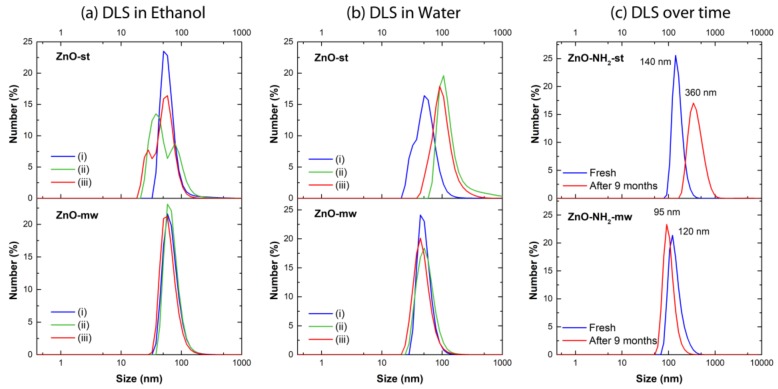
Dynamic light scattering (DLS) measurements in number % of pristine ZnO NCs obtained via traditional synthesis (ZnO-st, top panel) and via microwave route (ZnO-mw, bottom panel) in (**a**) ethanol; (**b**) water; (**c**) of functionalized ZnO particles (ZnO-NH_2_-mw and ZnO-NH_2_-st) in ethanol just after the functionalization procedure (solid line) and after nine months of storage (dashed line).

The colloidal stability of the nanocrystals synthesized by both methods and with or without amine-group functionalization was evaluated by DLS measurements, testing the behavior of three different batches in both ethanol and water and over time. Actually, to show the repeatability (or not) of the proposed synthetic approaches, three different batches are reported in three different color lines (blue, green and red) in Figure 5a,b. 

All the pristine nanocrystals obtained via microwave-assisted synthesis (ZnO-mw) result in being well dispersed in both ethanol and water media (Figure 5a,b, bottom panels). These ZnO-mw NCs show narrow size distributions, centered between 50 and 60 nm, and polydispersity indexes (PDI) of less than 0.2, characteristic of monodisperse samples. The nanocrystals synthesized via the traditional solvothermal approach (ZnO-st) present in contrast broader size distributions with higher PDI values (Figure 5a,b, top panels) than those obtained for the ZnO-mw samples, in both ethanol and water media. Furthermore, in some cases, the presence of different size distribution peaks is observed (Figure 5a, top panel). The lower colloidal stability of ZnO-st samples is even more evident in water (Figure 5b, top panel), where the PDI values are considerably higher and the size distributions of some of the tested batches are shifted towards bigger hydrodynamic diameter, indicating an aggregation of the sample (green and red curves in Figure 5b, top panel). A summary of PDI indexes and of mean diameter of number-weighted distributions is reported in Table 1.

The amine-functionalized nanocrystals show a good colloidal stability in ethanol, immediately after functionalization, as reported by the blue lines in Figure 5c (the top panel refers to the ZnO-st NCs, whereas the bottom panel to the ZnO-mw NCs). In the light of the clustering observed by TEM imaging and in order to verify the long-term colloidal stability and the shelf-life of NCs suspension for the biological application, the DLS measurements were also performed on the same batch of ZnO-NH_2_-st and ZnO-NH_2_-mw right after the functionalization procedure and after nine months of storage in ethanol. All the samples were subjected to 10 min of ultrasounds before the DLS analyses. The results (summarized in Table 1) indicate a reasonably good stability of ZnO-NH_2_-mw sample, with a mean hydrodynamic diameter that shift from 120 nm (blue curve) to 96 nm after nine months of storage (red curve, Figure 5c, bottom panel). In contrast, the ZnO-NH_2_-st NCs present a consistent increase of the mean hydrodynamic diameter (from 140 nm, blue curve, to 360 nm after nine months, red curve in Figure 5c, top panel) indicating an instability and a tendency to aggregation of NCs during the storage. These results also confirm what observed by TEM, despite the very different sample preparation, where the nanocrystals are dried on a copper grid in view of the TEM analysis, thus naturally tending to aggregate, whereas the DLS is performed in solution. 

**Figure 6:**
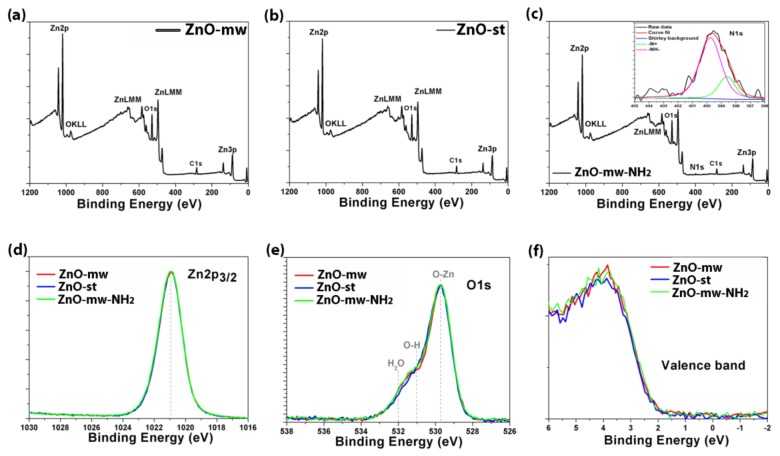
X-ray photoelectron spectroscopy (XPS) analysis: survey scans of (**a**) microwave-assisted synthesized ZnO nanocrystals (ZnO-mw NCs), (**b**) conventionally-synthesized NCs (ZnO-st NCs), and (**c**) amino-propyl functionalized NCs prepared by microwave assisted synthesis (ZnO-mw-NH_2_ NCs). Inset of Figure 6c reports the high-resolution spectra of the N1s region of the ZnO-mw-NH_2_ NCs. High-resolution (HR) curves comparing the three samples (ZnO-mw NCs in red, ZnO-st NCs in blue, ZnO-mw-NH_2_ NCs in green) for (**d**) Zn2p_3/2_ and (**e**) O1s regions. (**f**) Valence band (VB) signal related to the three samples.

Following the in-depth morphological and structural characterization, we have focused our attention on the physico-chemical analysis, starting from the rear surface, by means of the XPS technique, as reported in Figure 6. From the survey scans (see Figure 6a for the ZnO-mw NCs, Figure 6b for ZnO-st NCs, and Figure 6c for ZnO-mw-NH_2_ NCs) the relative atomic concentration (at.%) of each element is evaluated, as also listed in Table 2. The results on the ZnO-st-NH_2_ NCs are not reported in view of the similarities with respect to the other functionalized sample, ZnO-mw-NH_2_ NCs. Apart from Zn and O, we have also found C (due to the contamination from adsorbates) and N only in the functionalized ZnO-mw sample, as expected. In order to calculate the Zn/O ratio, we have subtracted from the O amount the components due to the bonds between C and O, after the deconvolution of the C1s high-resolution (HR) peaks (not reported). Therefore, it results that the microwave-assisted NCs have a Zn/O = 0.99, while the traditional ones have a Zn/O = 0.90. Furthermore, the functionalized microwave-assisted NCs show a Zn/O = 0.83. This means that there is an increase in the O amount in the rear surface of the latest two samples, which can be easily attributed to either the synthesis or functionalization procedures. In order to verify the oxidation state of Zn and O signals, the HR curves for each sample are compared. From the Zn2p_3/2_ curves (Figure 6d), no significant differences between the samples can be appreciated, since the three signals are perfectly overlapped and centered at the same binding energy (1020.9 eV), ascribed to the ZnO chemical shift [28]. Also the O1s region shows an almost perfect overlap between the three samples. From the deconvolution procedures (not reported) three components are obtained and are due to: O-Zn bond (529.7 eV), O-H bond (531.0 eV) and H_2_O residue (532.0 eV) as already reported in literature both theoretically [29] and experimentally [30]. Moreover, we have also checked the N1s region for the functionalized ZnO-mw sample, finding out that the experimental signal (reported in the inset of Figure 6c) is due to the imine group –N= (398.6 eV; 22.3 %) and amine group –NH– (399.7 eV; 77.7%). To complete the XPS analysis fully we have also acquired the valence band (VB) signal (see Figure 6f), which can give some more information regarding the DOS region, in order to have some more hints regarding the electronic band adjustment. From XPS measurements the valence band maximum (VBM) position, related to the Fermi energy level (EF), was extracted and corresponds to the 0 eV in our binding energy scale. The linear fit (not reported) of the descending part of each spectrum towards the EF, have given these values: 2.20 eV for the ZnO-mw NCs, 2.24 eV for the traditional ZnO-st sample and 2.14 eV for the amine-functionalized ZnO-mw one. These values are in accordance with that reported in the literature by Kamarulzaman et al. [31] for nanostructured ZnO particles.

To sum up, we can state that from XPS analysis the new microwave-assisted method produces NCs which are highly comparable, from the chemical and physical point of view, to those synthesized by the conventional solvothermal procedure.

### 3.2. Optical and Luminescent Properties of ZnO NCs

Owing to the fact that ZnO is one of the most excellent semiconductor materials, the prepared ZnO NCs are also characterized from the optical point of view. Actually, the optical and especially the luminescent properties of various ZnO nanostructures are well documented in the literature [32], both for spherical-shaped nanoparticle or nanowire form. In particular, ZnO NPs are reported to show good photophysical properties that, coupled conveniently with surface modifications, can be efficiently exploited as quantum dots in a biological environment for bio-imaging purposes [33]. In general, the sol-gel synthesis route and the large surface-to-volume ratio of the nanostructures can result in numerous defects on the surface of the ZnO NPs inducing a strong visible emission. In this regard, the optical properties of pristine ZnO NCs from both synthetic routes are investigated in this work. Furthermore, the literature reports about the influence of surface modification on the luminescence of colloidal ZnO nanoparticles [34,35].

**Figure 7:**
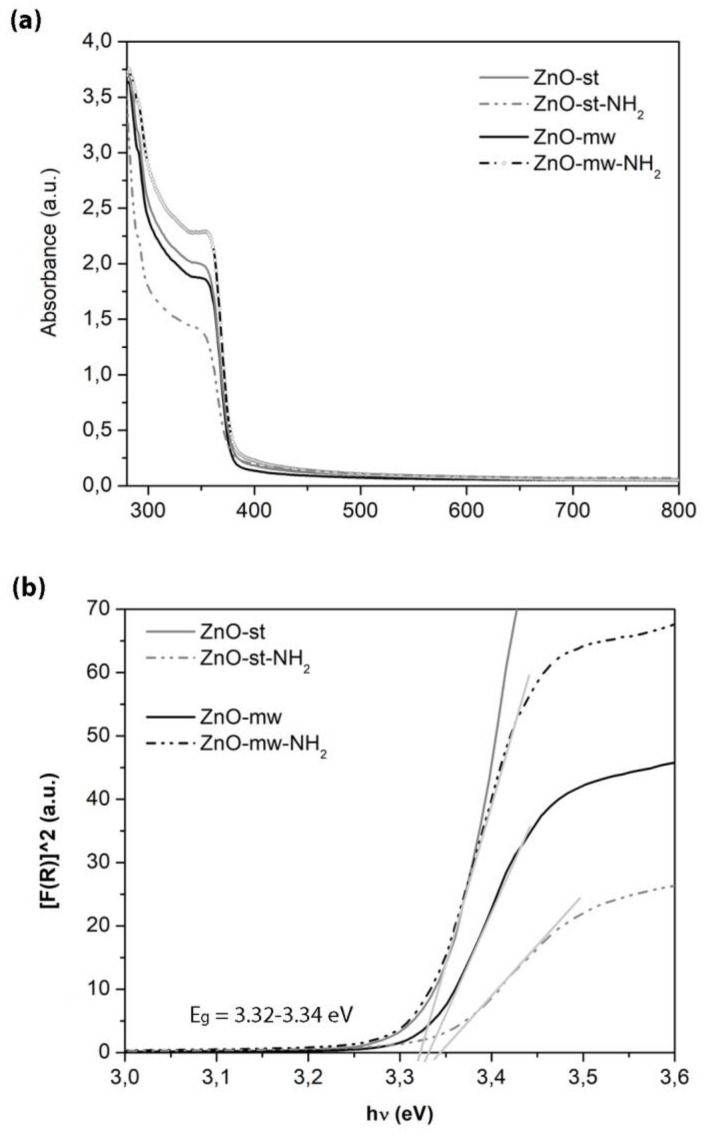
Characterization of the optical properties of pristine (solid lines) and functionalized (dashed lines) ZnO NCs obtained with the two preparation methods (conventional, in grey vs. microwave, in black). (**a**) Ultraviolet–visible (UV−vis) absorption spectra and (**b**) optical band gap (Eg).

UV−vis absorption spectroscopy was performed in the region between 300 and 800 nm, to point out the optical properties of the ZnO NCs and the related band gap (Figure 7). A comparison between the optical behaviour of NCs prepared by microwave-assisted synthesis and those synthesized by the standard one is provided, showing no differences between them. Actually, a typical and intense UV absorption is recorded for both kinds of nanocrystals in the region from 300 nm to 380 nm, characteristic of crystalline ZnO. Absence of absorption is recorded above 380 nm, showing full transparency in the visible region of the prepared NCs.

The optical band gap (Eg) of the samples was calculated using the Tauc’s method from the absorption spectra, as previously reported by some of us [36], see Figure 7b. According to this method, the plot shows a linear region just above the optical absorption edge. For the investigated samples, the resulting Eg is of 3.32–3.34 eV at room temperature, thus showing almost no significant variations among them. The differences in particle size and shape between the ZnO-st and ZnO-mw nanocrystals, cannot be appreciated in these spectra and the extracted band-gap values. In particular, the heterogeneity of size distribution observed for the ZnO-st NCs is still within a few nanometers, (i.e., from 6 to 20 nm, as estimated by FESEM) and the literature even do not report differences in UV-vis spectra from even broader sizes or shape variations in ZnO nanostructures [37].

**Figure 8:**
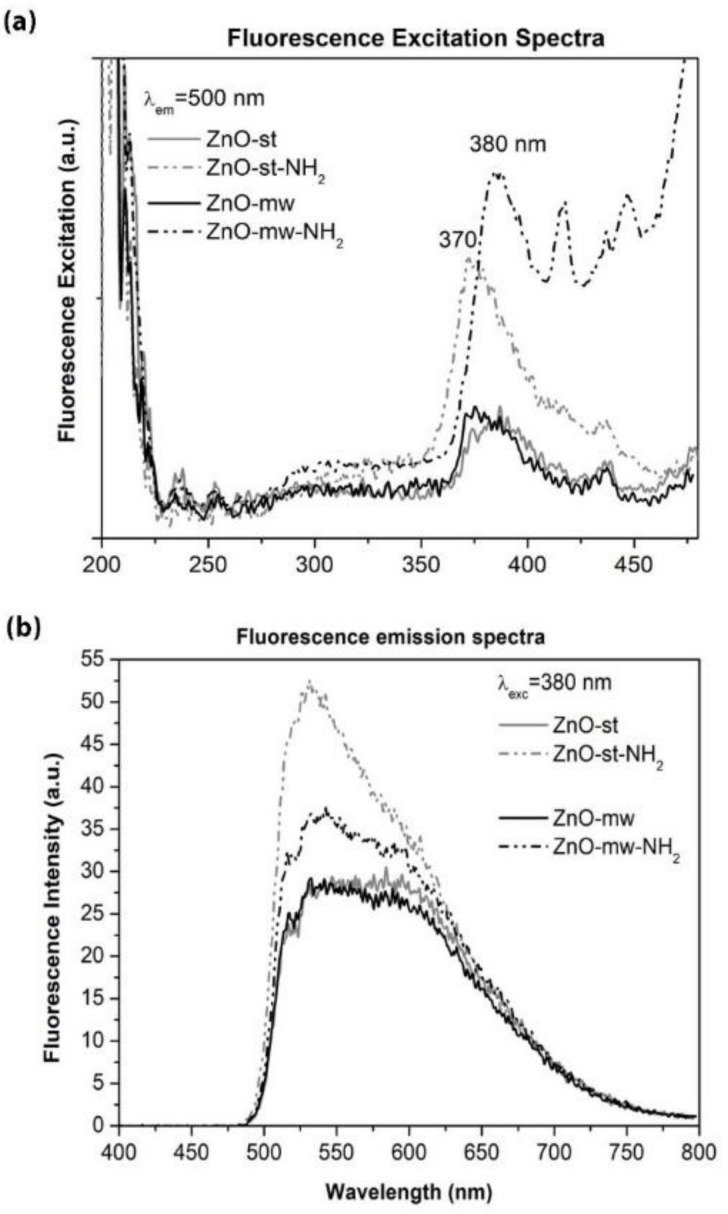
Luminescence properties of the pristine (solid line) and functionalized (dashed line) ZnO NCs obtained with the two preparation methods (conventional, in grey vs. microwave, in black). (**a**) Excitation spectra (recorded at the emission wavelength of 550 nm), (**b**) emission spectra (acquired at the excitation wavelength of 380 nm).

The fluorescence excitation and emission spectra are reported in Figure 8, comparing the behaviour of the ZnO NCs synthesized with the two preparation methods, conventional versus microwave-assisted one, and after their functionalization with amine groups, respectively. In the fluorescence excitation spectrum (Figure 8a), the highest excitation can be observed at around 380 nm and is similar for both pristine nanocrystals (solid curves), with a slightly higher excitation peak for the ZnO-mw NCs. This excitation can be ascribed to the direct exciton transition, i.e., the excited electron recombination with holes in the valence band (VB) or in traps near the VB [32]. Furthermore, a stronger intensity is recorded after surface functionalization with amino-propyl groups (dashed curves), in particular for the ZnO-mw-NH_2_ NCs. The enhanced fluorescence intensities of functionalized ZnO nanostructures were also previously described [34,35] and our results are in accordance with those in the literature. It is reported that amine-functionalized ZnO QDs further enhances the ZnO fluorescence by the well-known electron-donor effects of amine groups [35].

Considering the fluorescence emission spectra in Figure 8b, both kinds of NCs show a good and broad visible emission, from 500 to 700 nm approximately, when excited at λex = 380 nm. This visible emission is ascribed in the literature to crystalline defects, although these mechanisms are controversial and discussed so far. Actually, many point defects were suggested, including oxygen vacancies, oxygen interstitials, anti-site oxygen, zinc vacancies, zinc interstitials, and surface states [38]. As previously reported [39], there are two main mechanisms under discussion and considered responsible for the ZnO visible emission: (i) the recombination of an electron from the conduction band (CB) with a hole in a deep trap, and (ii) the recombination of holes from the VB with a deeply trapped electron.

It is again interesting to observe also in these emission spectra that the surface functionalization of the nanocrystals enhances their green emission, owing again to the electron-donor effects of amine groups.

Collectively, these results show that our ZnO NCs confirm previous literature data [32,39] about the good optical and luminescence properties of ZnO at the nanoscale. Furthermore, the presence of amine-functional groups leads to an enhancement of these luminescence properties, both in the excitonic emission and the green fluorescence emission. In addition, the surface functionalization with reactive amine-groups is useful for further biological modifications, i.e., anchoring of proteins and targeting ligands, as well as interaction with living cells, as reported below.

### 3.3. Cytotoxicity and Cell Internalization of ZnO Nanoparticles (NPs)

The cytotoxic effect and cell internalization rates of both conventional and microwave-synthesized ZnO nanocrystals was carried out on the amine-functionalized ones, i.e., ZnO-NH_2_ NCs, against the KB cancerous human cell line. This choice was made since the amine-functionalized NCs, as previously stated, can be easily labelled with fluorescent dyes for the detection at flow cytometry or further equipped with other functional biomolecules, as previously reported [25]. Furthermore, in a prospective approach to use them as nanoimaging tools, both ZnO-NH_2_ NCs have shown the highest luminescence properties.

**Figure 9:**
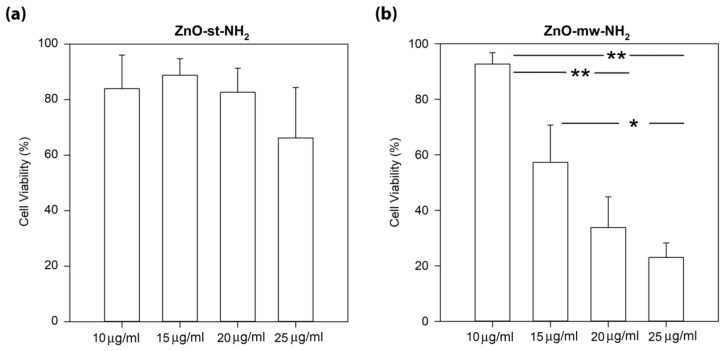
Comparison of the cell viability values of the (**a**) ZnO-st-NH_2_ NCs and (**b**) ZnO-mw-NH_2_ NCs at different concentrations toward KB cancer cell lines, detected by the WST-1 assay method. Cells were exposed in cell culture medium with different concentrations for 24 h. Results are expressed as the percent of cells viability compared to the control. The data are presented as the mean ± standard error (SE). ** *p* ≤ 0.001; * *p* = 0.05.

The WST-1 assay was used to quantify cell viability expressed as % of control. As shown in the left panel of Figure 9, ZnO-st-NH_2_ NCs did not exhibit any significant dose-dependent toxicity on cells. In details, the percentage of cell viability for 10, 15, 20 and 25 µg/mL concentrations result of 84% ± 12%, 89% ± 6%, 83% ± 9%, 66% ± 18%, respectively. Conversely, the results on ZnO-mw-NH_2_ NCs show a significant decrease of KB cancer cells viability in a concentration-dependent manner, as analysed by WST-1 assay after 24 h of exposure (right panel of Figure 9). Interesting evidence is the experimental toxicity range showed by the ZnO-mw-NH_2_ NCs; starting from 93% ± 4% at 10 µg/mL, the viability percentage decreases to 34% ± 11% at 20 µg/mL and to 23% ± 5% at 25 µg/mL. We note in details that the cell viability was significantly higher (*p* ≤ 0.001) for the treatment with 10 µg/mL than with those obtained with 20 and 25 µg/mL NCs concentration treatments. Furthermore, it should be evidenced that the 15 µg/mL concentration value could represent an interesting, effective and biocompatible cut-off. In addition, after exposure to ZnO-mw-NH_2_ NCs, KB cell line showed an IC50 value of 14 µg/mL.

The treatment of KB cells with either ZnO-st-NH_2_ or ZnO-mw-NH_2_ NCs for 24 h revealed that these NCs are non-toxic at 10 μg/mL and consequently we choose 10 μg/mL as a safe concentration for studying in vitro cellular uptake.

In flow cytometry the NCs load per cell is expressed as the number of events or intensity of the fluorescent signal associated with the labelled ZnO-NH_2_-Atto633 NCs. The integrated fluorescent signal from single cells is measured by side scattering and interpreted as either a NCs-containing cell or NCs-free cell [40].

**Figure 10:**
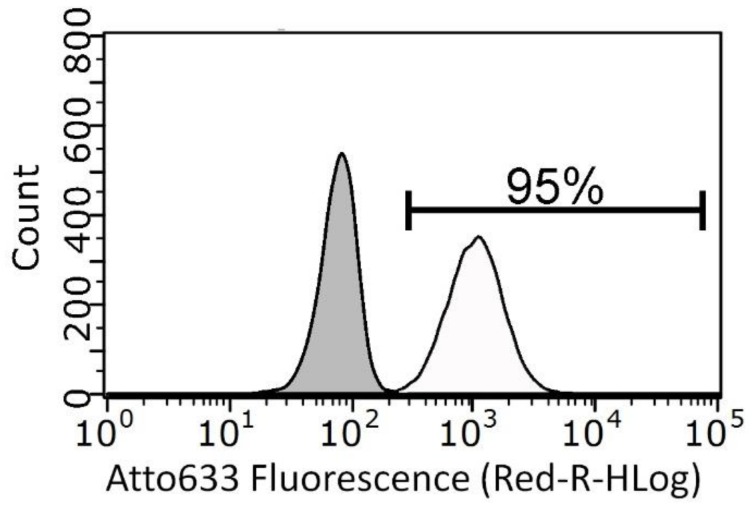
Representative flow cytometry curves of stained Atto 633 ZnO-mw-NH_2_ NCs uptake in KB cells. Grey area represents the untreated cells signal while the white one was obtained by cells incubated with 10 µg/mL ZnO-mw-NH_2_ stained with Atto633 for 24 h. Quantification of positively stained events, characterized by a shift in fluorescence intensity compared to untreated cells, was calculated setting a positive threshold beyond the grey area. Each condition is done in triplicate.

No statistically-significant differences appear between KB uptakes measured after 10 μg/mL of ZnO-st-NH_2_ NCs (74 ± 9) and 10 μg/mL of ZnO-mw-NH_2_ NCs (98 ± 0.6) treatments. It should be underlined that tests on cell treated with ZnO-st-NH_2_ NCs are not so reproducible as the one made using ZnO-mw-NH_2_ NCs, in fact, as shown above, standard error is considerably higher in the first case. In contrast, the uptake for cells treated with ZnO-mw-NH_2_ NCs is reproducible and well defined as reported in the representative flow cytometry curve showed in Figure 10.

## 4. Conclusions

In this work we report a new ZnO microwave-assisted solvothermal synthesis, optimized for biological uses. Homogeneous and spherical nanocrystals of 20 nm were obtained, with high reaction yields, and fully characterized from the physical-chemical point of view. By comparison with a chemically equivalent wet synthetic method we were able to evaluate their different biological behaviour, in terms of cytotoxicity and cell internalization in KB cancerous human cells.

X-ray diffraction, XPS and optical analysis demonstrated similar physical-chemical properties of the ZnO NCs obtained by the two different synthetic procedures, in terms of surface chemistry and electron band gap. However, we found substantial differences related to the hydrodynamic size, shelf-life stability (tendency to agglomerate in time) leading to a better reproducibility of biological test outcomes. Through a deep statistical analysis, it was in fact possible to estimate that the ZnO NCs, obtained via microwave synthesis, show more reproducible and reliable results.

These findings suggest that not only different preparation methods, but also similar procedures that generate particles with the same surface chemistry could drive biological responses to different ways. In particular, the ability to control and to obtain narrow and reliable NPs size distributions and highly stable behaviour in solution can be considered crucial factors in drive reproducible results, i.e., cytotoxicity and cell internalization tests. In fact, minor changes within the same synthetic route can alter both the shape and the size distribution. In our case, we demonstrate how the proposed microwave procedure is highly effective to better control and optimize the ZnO NCs morphology and size. Furthermore, we prospectively welcome the use of nano-sized ZnO particles with surface modification. We actually demonstrated that amine-functionalized NCs possess improved optical properties, useful for further bio-imaging applications, and allow future biomolecules anchoring, i.e., drugs, proteins or targeting ligands against cancer cells.

## Figures and Tables

**Table 1:** Polydispersity indexes (PDI) and average diameter of number-weighted distributions of pristine ZnO NCs obtained via traditional synthesis (ZnO-st NCs) and via microwave route (ZnO-mw NCs) in ethanol and water and of functionalized ZnO particles (ZnO-NH_2_-mw and ZnO-NH_2_-st) in ethanol just after the functionalization procedure and after nine months of storage.

	Ethanol			Water				Ethanol		
Sample		Av. diameter (nm)	PDI		Av. diameter (nm)	PDI	Sample		Av. diameter (nm)	PDI
ZnO-st NCs	(i)	65	0.28	(i)	56	0.52	ZnO-NH_2_ st NCs	Fresh	162	0.26
	(ii)	60 *	0.38	(ii)	155	0.36		After 9 months	410	0.25
	(iii)	53 **	0.38	(iii)	108	0.18				
ZnO-mw NCs	(i)	70	0.19	(i)	52	0.12	ZnO-NH_2_ .mw NCs	Fresh	144	0.11
	(ii)	71	0.14	(ii)	56	0.20		After 9 months	105	0.11
	(iii)	64	0.18	(iii)	49	0.26				

* Peak 1: 40 nm; Peak 2: 92 nm; ** Peak 1: 28 nm; Peak 2: 58 nm

**Table 2:** XPS relative atomic concentration (at.%) from survey data, together with Zn/O calculation in the last column. * Amount of oxygen, in atomic percent, that is associated with carbon (from survey data). ** Total amount of oxygen, from the survey, minus the amount of oxygen associated with carbon in atomic percent.

Sample	Atomic concentration (at.%)					
	C	O		N	Zn	Zn/O
ZnO-mw NCs	26.3	5.3 *	34.3 **	-	34.1	0.99
ZnO-st NCs	27.5	4.7	35.6	-	32.2	0.90
ZnO-mw-NH_2_ NCs	25.4	1.7	38.5	2.4	32.0	0.83
